# Exploring the Social Media on the Communication Professionals in Public Health. Spanish Official Medical Colleges Case Study

**DOI:** 10.3390/ijerph17134859

**Published:** 2020-07-06

**Authors:** Carlos de las Heras-Pedrosa, Dolores Rando-Cueto, Carmen Jambrino-Maldonado, Francisco J. Paniagua-Rojano

**Affiliations:** 1 Department of Advertising and Public Relations, Universidad de Málaga 29071 Málaga, Spain; lrandocueto@uma.es (D.R.-C.); fjpaniagua@uma.es (F.J.P.-R.); 2 Department of Economics and Business Administration, Universidad de Málaga, 29071 Málaga, Spain; mcjambrino@uma.es

**Keywords:** health communication, social media, public health, Spanish official medical colleges, stakeholders

## Abstract

The purpose of the study is to analyze the role that social media have on the practice of health professionals working in information and communication department of Spanish official medical college. Social media in health fields have experienced growing participation of users and are increasingly considered a credible form of communication. This paper examines the use of social media as communication tool by the Official Medical Colleges (OMC) of Spain. According to the National Institute of Statistics, in 2019 there were 267,995 registered medical professionals in the 52 OMC in Spain. This research is based on a qualitative methodological technique through semi-structured interviews, with the aim of identifying the profiles of the people who lead the information in the professional organizations of the OMC. Of the colleges, 73.07% participated. The findings show that information is essential for the OMC and most of them have at least one experienced communication professional. Social media are essential tool in their work and Twitter (87.5%) and Facebook (81.3%) are considered the most relevant social media according to their interests. These tools are believed to be very useful for informing, establishing relationships and listening to users.

## 1. Introduction

Nowadays, health communication plays an important role for citizens [1] and, therefore, contributes to social sustainability. Society is increasingly using the Internet in a bid to obtain health information, share experiences related to pathologic processes or find people with similar physical or psychological conditions [1,2]. Since information and communication technologies are being used in the field of health, terms such as e-patient or e-health are widely used, which is evidence of the increasing role citizens play in making decisions about their well-being [3,4].

Terrón [5] offers his perception from an anthropological point of view. In his opinion, the interest in health communication in a country like Spain has increased significantly due to the growing need for lifestyles that entail greater social well-being. This idea is reinforced in the feedback required by the supply and demand of information. Thus, interest in what is communicated grows as more information is offered [5,6].

The Internet has become the most important “loudspeaker” for patients’ expectations and demands. This fosters the emergence of associations that support patients’ rights to make their voices heard [5]. As a result, this makes patients feel stronger as they are members of a collectively supported platform on which they can express their needs and the needs of their environment.

Social media play a relevant role in this sense, with a progressive increase observed in terms of their use in the health field [3,7]. Factors such as the accessibility, immediacy or their potential to communicate bidirectionally with different audiences allow active communication [8]. Health centers are aware of the potential of social media and use them to promote interaction and collaboration between patients, relatives and professionals [9].

However, the democratization of information through social media [10] in the field of health means that social networks, blogs or mobile social media have developed peer communication with an increasingly participative audience, but above all, it has also made it more credible. Therefore, it confers greater communicational power to citizens and professionals in the sector when their messages reach a greater number of people [11,12].

These channels have become the preferred communication instruments for health corporations, by facilitating participation and collaboration with their stakeholders and allowing, thanks to a two-way communication, the control of the quality and efficiency of the institution [6,13], but also for the education of citizens with new healthy lifestyle habits [14,15]. It is also a key tool for communicating health alerts, the creation of networks of groups of patients with the same pathologies, or professionals for research purposes [12,16].

These real-time interactive information platforms provide a free online resource [17]. Therefore, another advantage of social media for the health sector is its low cost [7,18,19]. They are, therefore, a very effective two-way communicative tool for communication with stakeholders, such as sector professionals and patients [20].

The most popular health-related social media are those specifically intended for patients [1,21]. Nevertheless, other sectors of the population could also play a central role and benefit from the knowledge circulating on these networks if they became stakeholders of such information channels. Swan [1] suggests that agents in the health field who are meant to take care of patients, researchers and other agents involved in this area could take part more actively [21]. In the patient’s view, social media are the best instrument for real-time interaction, enabling the exchange of information and participation not only simply as patients or users, but also as groups or associations [22]. In the case of professionals, social media are used primarily for the dissemination of results, research, networking or teaching, among others [23].

However, this issue has disadvantages, the democratization of communication via social media entails, in some cases, a lack of veracity and information control [19]. Anyone may stir-up anxiety within society with their opinions or unconfirmed facts [6], as well as harmful criticisms or falsehoods directed at health professionals or health institutions [24]. It would not be ignored both the legal problems and lack of privacy, such as damage to professional image, violation of patient privacy, etc. [7,19,23]. In order to solve this problem, health institutions and professional organizations, such as Official Medical Colleges (OMC), have developed prevention guidelines and guides to good practices [7] aimed at protecting institutions in this area on a legal, clinical and organizational level [25,26,27,28]. OMC, together with health institutions, play a crucial role in developing recommendations for the use of social media. For this reason, the work of professionals with an expert profile in information and health is essential.

The choice of the official medical associations for this research is determined by the obligation that doctors must be registered to be able to practice the medical profession representing all the doctors in the country.

In detail, this work attempts to answer the following research questions:

RQ_1_: Are the official medical colleges in Spain valid interlocutors with their stakeholders?

RQ_2_: Do the communication professionals of the Spanish official medical colleges mainly use social media for their work as a source of information and verification?

RQ_3_: Do social media have any involvement in the agenda setting of the official medical colleges?

This study is structured as follows. It begins with a review of the existing literature focused on the importance of communication professionals and the use of social media in the health sector. Next, an analysis of the official medical colleges is carried out within the health field in Spain and analyzing the stakeholders with which it is related. Second, the methodology used is presented. This research work is carried out with a qualitative technique through semi-structured interviews with the communication experts of official medical colleges with the support of the Atlas.ti. Third, the most important research results are shown. The main contribution is a framework with strategic communication keys and the use of social media as an essential element of information. Finally, conclusion, managerial implications in health sector and limitations of the study are discussed.

## 2. Theoretical Background

### 2.1. Communication in Health and Social Media

Scientific literature regarding health communication highlights the necessity of the professionals practicing it to adapt to the changes brought about by the rapid invasion and evolution of social media.

With social media, access to information has changed, currently people do not rely exclusively on traditional or government media but trust the social media for essential information from the health sector [29]. Nowadays, social media such as blogs, websites or social networks such as Twitter, Facebook, Instagram, etc. are increasingly used by the population to acquire health knowledge [30].

It is necessary to design new strategies and challenges to cope with the apparition of a new style of network communication in real time [31]. Health professionals can make use of the new communication scenarios and their position as authoritative and credible sources in order to promote and defend health [32].

The professional practice of those who develop informative content for the media has been recognized for over a decade. As Arkin points out [33], in a study focusing on the American population, social media, as a leading source of health information, could potentially save lives in the event of a health crisis [29]. Nevertheless, those who develop informative content can also be alarmist and spread false information in the news or the coverage they offer.

Therefore, while the media selects the version of reality that it transmits and offers its own views on matters, it has the power to give this information the importance it considers appropriate [5]. It could be added that citizens search for increasing health communication, not only in what could be considered reliable sources, such as health professionals, but also in the media, so it seems necessary for the media to proceed responsibly [6]. Due to this fact, the media must offer true, transparent and coherent information [34].

Social media provide such health communication specialists with valuable information about patients’ experiences with which they can monitor public reaction to health problems. They also highlight the potential of such information for the development of health policies [35]. An example are medical blogs, which are frequently visited by the most important media outlets [36].

However, Leask, Hooker and King [32] also highlight how the role of specialists in charge of communication is losing relevance. This incurs a disappearance of the basic technical knowledge that is necessary to transmit health communication correctly. One of the new challenges is developing tools to verify contents, given the risk of spreading inaccurate or false information generated by the ever-increasing speed with which information emerges [31].

Rumors, conflicting news and speculation are characteristics of the messages circulating within social media. According to Hermida [37], it is the responsibility of media professionals to select, contextualize and verify the enormous amount of information. In this sense, it is not conceivable that the health communicator is dependent on sources, funders, or other informants with certain interests to reliably inform society and not be detrimental to the well-being or the quality of life of society [38]. Therefore, ethics and responsibility at work, as well as a commitment to society, are key elements of the communicator. This is how better care is offered to the population [39]. This work is reflected when organizational interests are transferred in order to reinforce the welfare or quality of life of citizens.

However, the veracity of the information lies with the communication professional, the determination of correct information now becomes complicated due to, among other aspects, the vast amount of information generated by social media. Knowing how to use them for professional purposes is no easy task and a new type of reasoning is required. In spite of this, the authors both defend this new scenario, saying that the communicative potential of social media is “far from being negligible” [40] (p. 67).

As well as verification and credibility of the information spread through social media, other aspects of social media that the studies under analysis expose are the effects that these produce in professional practice [41]; the use that professionals make of them [42] and their importance for communicators or the weight they bear regarding information [43].

Since McCombs and Shaw proposed the theory of agenda-setting in 1972 [44,45] to explain how the media influence the shaping of public opinion, its application to research has been intense and fruitful. In the process of establishing the news that attracts the attention of the audience, each media has tried to play a differentiated role [46]

The change of the hierarchical structures regarding the organization of the information by the spaces of conversation, connectivity and the creation of a community that have provoked social media is one of the changes highlighted by Hermida, Lewis and Zamith [41] in the exercise of communication professionals who work on social media. There is no longer a single paradigm for the structure of news as proposed by Almaguer [46], but there are many different ways of developing content. Even if corporations seem particularly interested in social media as a vehicle to market news content, increase traffic to their websites, and strengthen relations with the customers, communication professionals, on the other hand, mainly use social media to talk about what they are working on or share opinions or ideas. This implies that the content provided by corporations does not enrich the media agenda. On the contrary, according to these authors, news that refers to social media as the source of information is rare or infrequent [42].

Lariscy et al. [43] not only put the emphasis on the attention that corporations should expend in terms of the content that is disseminated through social media, in addition, they point out that the task of communication or public relations professionals are to closely monitor the information issued from the entities for which they work and possibly involve those who are the originators of the content [47], considering that social media contribute to the construction of the agenda setting [48,49,50].

Different studies highlight the potential of hospital social media and how society can profit from them. In this way, Shepherd et al. [51] place these in a favorable and expanding environment, which favors personal relationships. De la Peña and Quintanilla [52] (p. 495), describe the role they play for citizens as “a virtual community where they can find stimulation, get answers to specific questions related to health and a place to share success stories” and Koteyko et al. [53] add that this tool has great potential to promote initiatives.

To the field of hospital information, the content producers would be health professionals and management and service professionals, together with users of the health system, patients and family members, among others.

Influencing the importance of information supervision, social media is considered as an effective instrument to promote communication between public administrations and different stakeholders [48,54]. Specifically, in the sector in charge of communication through social media within the field of specialized health care, scientific publications that focus on the practice of communication professionals specialized in health are scarce [49,50,55].

However, as well as these publications that underline the benefits of health communication by means of social media, there are others that focus on their harmful effects or potential dangers. Among these dangers are the loss of privacy or security regarding shared information [50,56] and the lack of specialized training, both in health and in the management of social media among communication professionals [57,58].

### 2.2. Official Medical Colleges (OMC) in Spain

In many countries, registration in the medical association is legally mandatory for all doctors who practice medicine temporarily or permanently. Thus, in Europe, as examples, there are the *Conseils de l’Ordre des Médecins* in France, the *Fédération des Médecins Suisses*, the general medical council in the United Kingdom, the *Ordre des Médecins* in Belgium or the *Colegio Oficial de Médicos* in the case of Spain. The first regulations in Spain of the official medical colleges date from April 12, 1898, but it is not until the Royal Decree of May 28, 1917 where the compulsory registration in the college to practice the medical profession is definitively approved.

Professional colleges play an essential role. This relevance of professional services lies in the protection of the rights and interests of citizens who receive them.

Throughout diverse regulations on professional associations in Spain, the sector has undergone different transformations, but maintains the same structure that it originally had. The law defines professional associations as legal public corporations, protected by the law and recognized by the State, with their own legal personality and full capacity for the fulfilment of their purposes, which empowers them as entities that represent and defend the profession that each college has and points out the obligatory registration for the professional exercise which is regulated by law.

In the present case, the Spanish official medical college promotes the scientific work of doctors and connects this collective with patients and society. Most importantly, its ethical code committees ensure that health institutions adhere to the norms.

There are currently 52 official medical colleges in Spain with a territorial structure. The number of doctors registered at the official medical colleges have been increasing year-by-year to reach a total of 267,995 in 2019, as represented by Table 1.

**Figure 1:**
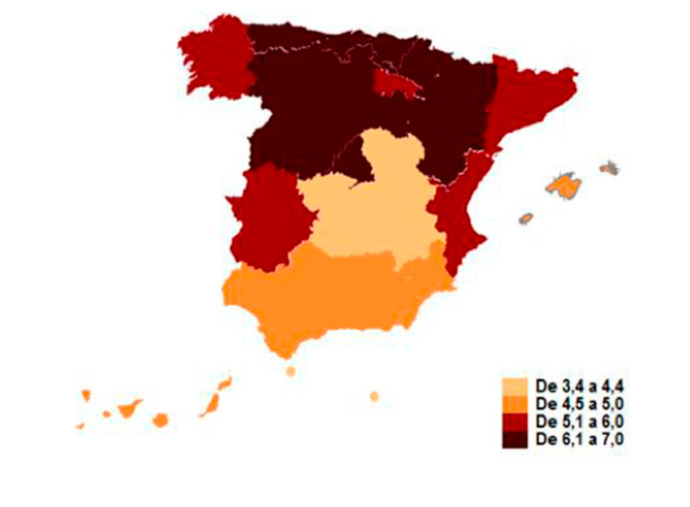
Registered medical professionals in 2019. Quota per 1000 habitants. Source: Instituto Nacional de Estadística. 2019 [59].

The number of registered medical professionals per 1000 inhabitants is 5.66 on average. By autonomous communities, the representation is as follows (Figure 1).

#### Official Medical Colleges and the Relationships with Stakeholders

The situational theory of Grunig’s audiences [60] identifies stakeholders as groups formed by people who unite them with a problem or end of a similar nature and of which they are aware and so they group together to adopt a proactive attitude focused on action, in its attempt of resolution.

The professional management of communication according to Grunig and Hunt [61] identifies three weaknesses and three strengths. The weaknesses are: (a) the saturation of the communicative channels due to the lack of well-documented and orderly information that ends up becoming irrelevant to the public, (b) frequently this information overshadows the true relevant facts of interest and (c) it ends up generating distrust in interest groups because of the sense of deception. The strengths are: (a) it approaches and sensitizes the entities with their stakeholders, by establishing a more dynamic and bidirectional communication, (b) it acts as a clear and concise spokesperson for issues of public interest and (c) it promotes the knowledge of the various stakeholders through information in formal and informal communication media.

For Ruiz de Azua [62], health communication management requires an open and empathetic communication style that generates public trust. It is most effective when health professionals try to stimulate the population to take positive action or abstain from a harmful act. However, trust is essential, public mistrust of other stakeholders such as health experts and representatives of health institutions can increase for a variety of reasons, one of the priorities being access to conflicting information collected in the social media.

Mainardes, Raposo and Alves [63] emphasize the importance of identifying and aligning stakeholders with the strategic objectives of their institutions, given that they themselves play a vital role in the development of management strategies. Therefore, any serious and reputable entity needs to develop and implement its strategy through specific expert structures created with the objective of directing relations with its audiences. Due to the powerful position and performance they entail [64], proactive care of these can become a huge advantage for the reputation of the official medical colleges.

**Figure 2:**
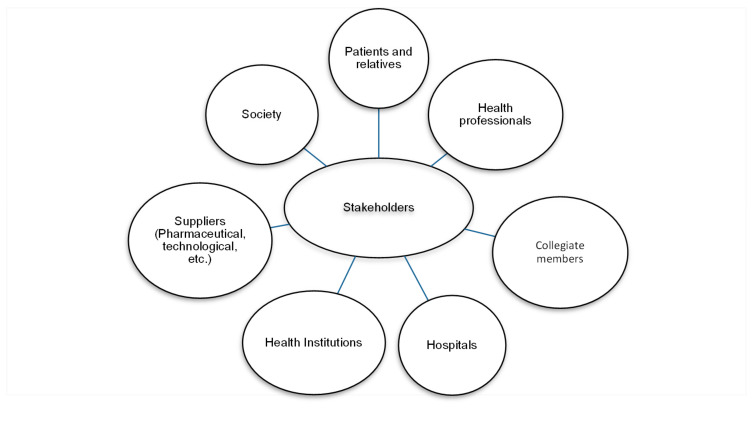
Stakeholders of Spanish official medical colleges. Source: Adapted from Freeman [66].

Therefore, an express knowledge of the stakeholders and the relationship that must exist between them is essential [65]. Next, the following Figure 2 establishes the public map that relates to the official medical colleges. Beginning with the main audience to which they are directed as their own members are, guiding them in good ethical practices, in technological advances and in health research. Continuing with healthcare professionals such as nurses, psychologists, therapists, physiotherapists, etc., including the providers where pharmacists and health engineers will be highlighted and ending with patients, their families, health institutions, hospitals, government or the media and society itself in general. In short, once the map of the stakeholders is represented, the prioritized objectives will be established in order to design the communication strategies and policies appropriate to each one of said audiences.

For this reason, this study focuses on the degree of knowledge that such professional college have regarding current communication tools, among which social media plays an influential role that affects the information that reaches its stakeholders. The Spanish official medical colleges must continue transmitting reliable information using language that is appropriate for the whole population. Therefore, the existence of specialized communication professionals who are at the vanguard of communication languages, mechanisms and strategies is so important.

## 3. Methods

A qualitative method through semi-structured interviews has been applied with the aim of delving deeper into the use of social media by communication professionals specializing in health. The choice is relevant as the main contribution of this qualitative study is to explore and understand some features of the communication of these colleges.

Qualitative research (QR) is useful for analyzing and understanding interpersonal relationships, behavioral experiences and variables such as opinions, perceptions, motivations and attitudes [67,68]. These variables are of interest in the field of communication. Moreover, the flexible and eminently inductive nature of QR is particularly suitable for seeking explanations for communication phenomena. Atlas.ti is the tool used to systemize the data and provide the desirable insights.

However, given the novelty of the issues being discussed and the need to ensure that the views of the most important actors were obtained, the flexibility of semi-structured interviews greatly outweighed the limitations on statistical analysis that would result [69,70,71,72]. In fact, flexibility both in designing and refining the interview guides and in actually conducting the interviews is probably the most important key to success in using this technique. This kind of interviewing also allowed the research to explore some of the underlying motives more directly [73]. Hence, semi-structured interviews using a deductive approach [74] were chosen in order to allow the participants a degree of freedom to explain their thoughts and to highlight areas of particular interest and expertise that they felt they had [75], as well as to enable certain responses to be questioned in greater depth, and in particular to bring out and resolve apparent contradictions.

The research team developed a protocol for semi-structured guides, that included five phases: (1) identifying the prerequisites for using semi-structured interviews; (2) retrieving and using previous knowledge; (3) formulating the preliminary semi-structured interview guide; (4) pilot testing the guide; and (5) presenting the complete semi-structured interview guide.

The interviews were carried out by the authors.

All 52 of the OMC in Spain were contacted, addressing the person in charge of communication or, whenever this information was not publicly available, addressing the Dean as the head representative. Of the OMCs, 38 showed interest in participation; they represented 73.07% of the total. The high participation of the OMC allowed to obtain information from a highly representative group. For this reason, it was considered interesting to include some statistical data, which are reflected in tables.

The guide included three predefined categories. At first in order to know the profile the professional who lead the communication in the official medical colleges, they were asked some data about their functions and tasks, as well as the labor seniority, based on a validated tool by European Monitor Communication studies [76]. It was also considered important to know first of all aspects related to gender and age and especially those related to their work situation and whether there were communication cabinets in the OMC and whether the professionals responsible for them were experts with communication training.

The first category was regarding the involvement of the official medical colleges’ stakeholders. The role of health institutions, health professional, hospital groups, patients and family members or society were discussed. This debate helped us to have enough knowledge to map the stakeholders with whom they were dealing.

Second, we were interested in knowing the importance of communication professionals in the OMC and their use of social media for their work. The main reasons why they use social media in their professional routine (as a source of information, to interact with its stakeholders, as a communication channel, to establish professional relationships, to search for stories or to contrast information) were discussed in the interviews.

Finally, the third category was related OMC as producers of heath information. Social media has brought opportunities and challenges in health field, so we were interested about how social media contribute to the construction of agenda-setting in the health sector.

## 4. Results

Going into detail concerning with the qualitative technique, it is shown a descriptive analysis of the participant in order to present a general view of its make-up and highlight the main relationships between variables and their significance for the population (Table 2).

Both their years of work experience in the health communication sector and their activity in social media validate their extensive knowledge in the field.

Over half of the interviewees, is between 30 and 39 years old, whereas the rest is over 40. All of them were part of the working-age population when social media appeared, according to the mapping of birth with the evolution of social media that Boyd and Ellison made in 2008 [77,78].

The number of years of professional experience is also worthy of attention. Over half of the professionals have worked for over ten years and the rest have worked for between five and ten years. This is significant if it is considered that specializing in the health sector, which since the 90 s has been affected by a “superabundance of information” [79], implies years of professional experience in order to provide quality information.

As it was indicated before three categories were predefined. Table 3 summarily describes the themes obtained of participants.

**Figure 3:**
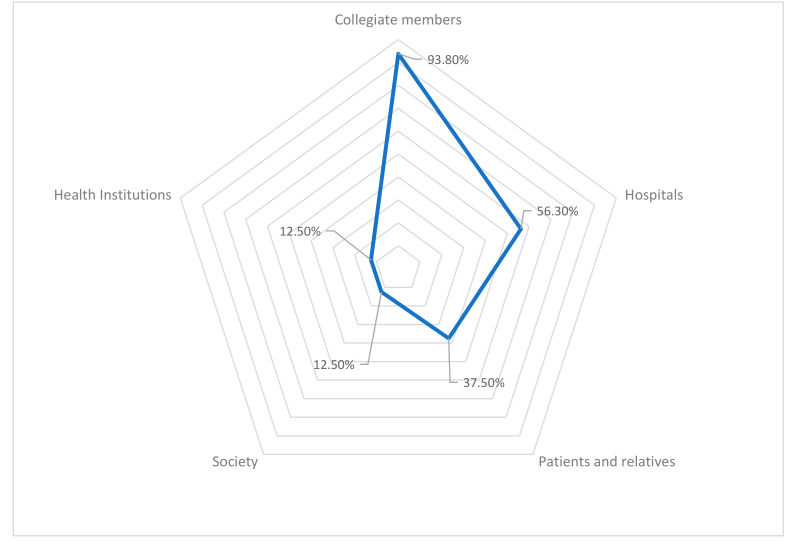
Official medical colleges (OMC) relations with their primary stakeholders. The percentage indicates the relationship with each stakeholder. Source: In-house elaboration.

Regarding RQ_1,_ the experts highlighted that the most important stakeholders for the OMC are their own collegiate members that corresponds to all medical professionals enrolled in their district, with which they maintain regular bidirectional communication. When considering that they should be informed of all the concerns and investigations that are carried out by all health professionals. Followed by public and private hospitals. Patients and their families showed scarce representativity. Finally, society in general and health institutions were the groups with the lowest score (Figure 3). Comparing Figure 3 with the theory of Freeman’s stakeholders seen in Figure 2, the only groups that are not represented are the suppliers (pharmaceutical, technological, etc.) and other health’s professional (nurses, physiotherapists, etc.).

Regarding question RQ_2_, all official medical colleges participants in this investigation have an organized communication department, in which one to three people work—either specialists in the field and graduates in Journalism and/or Public Relations. In addition, these are organized in a communication network of professional medical colleges, to exchange experiences, and even organize an annual congress to present and share their initiatives. Recently, they have dedicated these conferences to social media strategies, and they have also worked in the fight against the dissemination of fake health news or crisis communication.

In terms of usability, the recurrent use of social media as an instrument of work exposed in the scientific literature referred to is corroborated in the results obtained in the interviews. The fact that all respondents use social media in their work may reflect the challenge of incorporating social media. Most indicated that their usage times are growing annually. The following table reflects the number of hours/days of social media use to collect or disseminate information (Table 4).

It is important to highlight that, despite the fact that 18.8% of those interviewed consider that social media hinders their work because excessive information published results in intoxication of the content, most of the respondents think that social media make their job easier and lead them to other sources of information (Table 5).

Nevertheless, social media have become a source of information that is relevant or very important for most professionals (Table 6).

It was highlighted by respondents that content generated by unofficial sources or by users was not sufficiently rigorous.

With regard to health-related accounts. All of them visit the corporate social media of hospitals and/or health institutions and evaluate their content as relevant (Table 7). None of the respondents considers the content found on hospital or health institutions social media to be “not important at all”.

**Figure 4:**
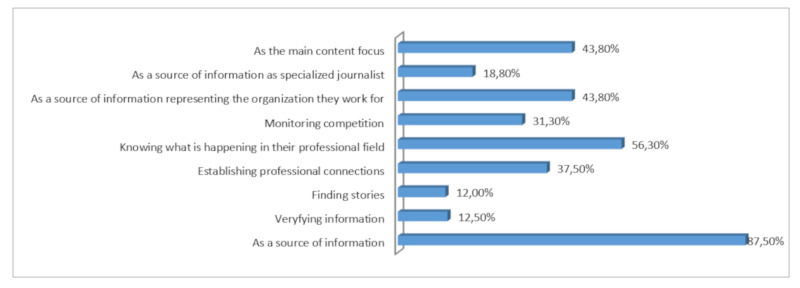
Main aims in the use of social media. Source: In-house elaboration.

The participants use social media as a “source of information”, which is one of the main aims for using them. In addition, the respondents highlight that this motivation is followed by keeping up with what is happening in their professional field. The aims that follow in the ranking of importance emerging from the interview are “as a source of information for the organization they work for”, “as the main content focus”, “establishing professional connections”, “monitoring competition”, “as a source of information as specialized journalist”, “verifying information” and “finding stories” (Figure 4).

In the case of social media as a source of information, the difference between the percentage of Twitter and Facebook users increases, so that Twitter emerges as the top social media chosen by specialized professionals. All of them point out social media as the main channel in the future.

In particular, out of the social media that they use, specialized health professionals in Spain resort most often to Twitter and Facebook (Table 8).

All of the interviewers visit social media that are managed by health institutions, follow by health professionals accounts and researchers. Almost a third of the participants mention patient collectives that also communicate by social media.

In terms of credibility, the most credible accounts are those of health institutions, to which 87.5% refer. They are followed by the accounts of health professionals and researchers in the health sector. If the public and private hospitals are considered separately, the public hospitals accounts enjoy greater credibility than those in the private hospitals. The patient collectives accounts are the least credible. The accounts of citizens expressing themselves on the topic of health and who do not belong to any association do not enjoy any credibility (Table 9).

The argument that the respondents give for their answers is scientific rigor, which is considered to be greater in the case of health institutions. The communication on these social media is contemplated dependable because it is supported by the institution they represent and the professionalism of is specialists.

It is necessary to point the low level of credibility in public and private hospitals. The professional proposes changes in hospital social media, such as contents that are not biased according to institutional interests, more information about health in general and about health promotion in particular that can be communicated to the citizenship. Another suggestion has been to give a relevant role to the hospital professionals as co-creators of contents.

In their responses, communication professionals of OMC do not coincide in their opinions about how institutions avoid managing communication through social media in the event of a health crisis. It is widely recognized in the literature that social media offer immediate circulation of messages and this makes them valuable for such professional communication.

The speed with which information can be spread through social media in emergency situations, catastrophes and socially critical events which are dangerous for public health makes them essential for professionals specializing in health.

With respect to RQ_3_, the participants think that social media is a useful tool for establishing bidirectional communication with their registered doctors, health institutions, hospitals and patient associations. Likewise, the contents generated in their social media accounts have scientific rigor that is very useful for society.

The professional of OMC defend that unlike the beginning of the decade, when the participation of the official medical colleges in Spain in social media was insignificant, today its activity is remarkable. This can be noticed in the significant increase both in the number of followers of their social media and in the volume of information they generate. The voice of the professionals of these entities in social media acquires special relevance in order to promote accurate information that preserves the quality of life and well-being of citizens.

The interviewees consider that official medical colleges have the capacity and the authority to become the voice for health institutions in their role as official sources in matters of health, using its own social media accounts at times when citizens claim a continuous supply of information.

## 5. Conclusions

However, organizations and professional associations in the health field are increasingly examining the potential of social media to allow members to share knowledge and engage their publics [54], these activities in official medical colleges were under researched [5,24].

Spanish official medical colleges, with a total of 52 locations distributed in each of the Spanish provinces and with 267,995 registered doctors, are considered one of the most important health institutions in the country.

Unlike other health institutions, communication is essential for the official medical colleges in Spain. Therefore, they have communication cabinets with professionals specialized in communication with extensive knowledge in the work they develop as part of their staff with more than five years’ experience. Their professionals have degrees in journalism and/or public relations and are mostly women.

They have shown a strong relationship with stakeholders such as health professionals, health institutions, hospitals, patients and their families and society in general. Consequently, it can be stated that RQ_1_ can be answered in the affirmative, the official medical colleges represented by their communication experts are valid interlocutors with their stakeholders.

OMC’ professionals analyzed and verified the social media information of their stakeholders. Social media are a valid source of information for aspects related to new developments in the field of health.

All the communication professionals consulted use social media for their work. Twitter and Facebook are the most widely used; Twitter being the most popular (87.5%), followed by Facebook (81.3%). It coincides with Ahmed [80], that points out the most important social media for health conversations is Twitter, where it is also possible to extract tweets for academic research purposes. Through social media there can be the possibility of key debates and relationships with other stakeholders related to health topics.

The credibility of the corporate social media of health institutions and hospitals, based on the veracity of the information they publish, is well appreciated by communication professionals at the official medical colleges of Spain. However, it is significant that a percentage of the participants considered that these hospital contents are not sufficiently complete to serve their interests, and therefore are unsatisfactory.

The value afforded by the communication established by hospitals through their corporate social media has been useful in analyzing the influence of these contents on the daily practice of professionals specialized in health working in the communication departments of the Spanish official medical colleges. The contribution of these professionals, as intermediaries between the health institutions and the society in the construction of reality, has revealed strengths and weaknesses of hospital social media.

With the purpose of bringing the professionals on both sides closer together and making their relationship more successful those in charge of hospital communication, would must rise to the challenge of active listening to the communication specialists of the Spanish official medical colleges.

This finding coincides with Castilla [24]. Those in charge of setting the health agenda have expressed that in such circumstances, they would become more permeable to information coming from hospital social media, which would play a more significant role as relevant sources of information. This fact would reverse the percentages of credibility obtained in this respect.

These professionals find social media relevant, they know their potential and value the volume of information that they can access through them, even if their contents make up only portion of their agenda-setting. In this sense, most professionals consider that social media are one more tool that they can use in their work, but few of them include them how only as a source of content. Fulfilling research question three.

There is no total consensus among those interviewed that social media is the best channel of information in the context of health crises. For them there is much noise on social media with unconfirmed or fake news.

Most of the experts suggest social media as the main communication channel with stakeholders in the future.

### Limitations and Future Research Lines

First, since this is an exploratory study of the behavior of official medical colleges, it is not possible to measure development in the medium term, and a lengthier study is needed in order to advance. Other qualitative research techniques such as content analysis on social media could be integrated in order to perform more in-depth analysis. Second, research has only focused on Spain, so further empirical research should be conducted in different regions, which would be of interest to make comparisons with other countries. The limitations identified are the basis for the design of future lines of research.

## Figures and Tables

**Table 1:** Number of doctors registered at the Spanish official medical colleges. Series 2015–2019.

	2015	2016	2017	2018	2019
Doctors	242,840	247,958	253,796	260,588	267,995

Source: Instituto Nacional de Estadística. 2019 [59].

**Table 2:** Demographic factors.

		Frequency	Percent
**Gender**	Male	12	32.70
	Female	26	67.30
**Age**	26–29	0	0
	30–39	22	57.90
	40–49	8	21.05
	50–65	8	21.05
**Employment situation**	Communication Cabinet OMC	35	92.10
	Freelance	3	7.90
**Job experience**	Less to 5 years	0	0
	5 to 10 years	17	42.10
	More than 10 years	21	57.90

**Table 3:** Main Themes.

Theme	Description
The role of OMC as interlocutor with stakeholders.	References that coincide in the same stakeholders, focusing mainly on the collegiate members, then in the hospitals and health institutions and finally in the patients and society. All OMC communicators confirm that there is bilateral communication with them.
Social media as a work tool for this professional.	References indicate that they use social media to learn about advances in the health field, connection with other health institutions, professionals and verification of fake news.
The importance of social media in the communication strategies of the OMC and the implementation of its agenda setting.	References to social media as a useful tool for bidirectional communication in the preparation of agenda setting.
	Social media is changing communication pattern, although it is recognized social media is not the only source used for the realization of the agenda setting
	There is no total agreement among those interviewed that social media is the best channel of information in the context of health crises.

**Table 4:** Use of social media per day (%).

Communication Experts	Use of Social Media per Day (%)
Less than 1 h	23.80
Between 1 and 2 h	51.20
Between 2 and 5 h	13.50
More than 5 h	11.50

**Table 5:** Usefulness of social media for daily work.

Usefulness of Social Media for Daily Work	Utility (%)
Make work easier and increase the possibilities for information sources.	75.00
Hinder their work because there is too much information and sometimes intoxication.	18.80
Increase the workload and time spent	6.20

**Table 6:** Social media as an information source in your work.

Social Media as an Information Source	(%)
Very important	62.50
Important	25.00
Low importance	12.50

**Table 7:** Importance of the social media related to the health sector.

Importance of the Social Media Related to the Health Sector	(%)
Very important	12.50
Important	56.20
Not so important	12.50
Neutral	18.80
Not important at all	0

**Table 8:** Social media used most frequently.

Social Media	Use (%) The Percentage Indicates the Level of Use with Each Social Media.
Facebook	81.30
Twitter	87.50
LinkedIn	12.50
Pinterest	0
Google +	18.80
Specialized scientific and health networks	18.80
Instagram	6.30

**Table 9:** Health-related social media.

Accounts Social Media	Consult (%)	Credibility (%)
	The Percentage Indicates the Relationship with Each Account Social Media.	
Health institutions	100.00	87.50
Health professionals and researchers	56.30	31.30
Public hospitals	56.30	18.80
Private hospitals	50.00	12.50
Patient collectives	31.30	12.50
Citizens	0	0
